# Does hunger promote the detection of foods? The effect of value on inattentional blindness

**DOI:** 10.1007/s00426-021-01480-y

**Published:** 2021-02-06

**Authors:** Dennis Redlich, Daniel Memmert, Carina Kreitz

**Affiliations:** Institute of Training Science and Sport Informatics, German Sport University Cologne, Am Sportpark Müngersdorf 6, 50933 Cologne, Germany

## Abstract

Although human perception has evolved into a potent and efficient system, we still fall prey to astonishing failures of awareness as we miss an unexpected object in our direct view when our attention is engaged elsewhere (inattentional blindness). While specific types of value of the unexpected object have been identified to modulate the likelihood of this failure of awareness, it is not clear whether the effect of value on inattentional blindness can be generalized. We hypothesized that the combination of hunger and food-stimuli might increase a more general type of value so that food stimuli have a higher probability to be noticed by hungry participants than by satiated participants. In total, 240 participants were assigned towards a hungry (16 h of fasting) or satiated (no fasting) manipulation and performed afterward a static inattentional blindness task. However, we did not find any effect of value on inattentional blindness based on hunger and food stimuli. We speculate that different underlying mechanisms are involved for different types of value and that value manipulations need to be strong enough to ensure certain value strengths.

## Introduction

In our daily life, we sometimes do not notice what appears in our line of sight, which is a well-established phenomenon in the scientific literature, termed as inattentional blindness. Inattentional blindness occurs when an observer fails to notice an unexpected object while being engaged in a resource-consuming task (Mack & Rock, 1998; Most, Simons, Scholl, & Chabris, 2000). Such failures occur in everyday life and can be of minimal importance (e.g., failing to notice a square on a computer screen or a unicycling clown on campus; Hyman, Boss, Wise, McKenzie, & Caggiano, 2010) or can have tragic consequences (e.g., failing to notice a tumour in medical diagnostics; Drew, Võ, & Wolfe, 2013).

While the likelihood that an unexpected object is noticed has been investigated extensively in the context of individual differences (Bredemeier, Hur, Berenbaum, Heller, & Simons, 2014; Kreitz, Furley, Memmert, & Simons, 2015) and situational factors (Kreitz, Furley, & Memmert, 2016; Most et al., 2001), the value of unexpected objects has received relatively little research attention. Previous studies have shown that evolutionarily predetermined value as animacy (Calvillo & Jackson, 2014) or threat (New & German, 2015) and overlearned value as the word “STOP” or one´s name (Mack & Rock, 1998) indeed affect the susceptibility to inattentional blindness. In contrast, monetary value learned incidentally over a short period of time does not seem to affect whether this failure of awareness occurs (Redlich, Schnuerch, Memmert, & Kreitz, 2019).

Li et al. (2015) investigated the effect of value related to ice cream on the susceptibility to inattentional blindness by comparing students with high or low levels of ice cream craving. The value of ice cream might be learned through a combination of processes over different periods of time here: the momentary state of appetite for ice cream and the general overlearned value for ice cream. The authors found significantly higher noticing rates for unexpected ice cream stimuli when students had a high level of ice cream craving. However, Li et al. (2015) used a quasi-experimental design as the ice cream craving level was not experimentally manipulated. Also, they focused on specific circumstances as female students and ice cream stimuli. Consequently, one might question whether the effect of value caused by craving can be generalized over sexes and different food stimuli. Hunger might be especially suited to further investigate the effects of value on inattentional blindness as it can be temporarily and experimentally manipulated (short-term individual difference) but roots in strong evolutionarily predetermined value (long-term value).

Seeking and consuming certain foods and associating food stimuli with a specific value are motivated by the rewarding effect of satisfying hunger (Berridge, 1996). This value is strongly affected by time as it increases when we are hungry and decreases when we have just eaten (Cabanac, 1971). Support for cognitive effects of hunger has grown in recent years: Hunger has been found to increase selective attention (Mogg, Bradley, Hyare, & Lee, 1998), improve the memory advantage for food stimuli (Morris & Dolan, 2001), limit attentional shifting (Piech, Hampshire, Owen, & Parkinson, 2009), and increase attentional capture as demonstrated with a stronger attentional blink for food pictures (Piech, Pastorino, & Zald, 2010). This effect of hunger on cognitive processes might be explained by adjustments in one’s attentional set towards food-related stimuli. Fittingly, Higgs (2016) hypothesized that merely thinking about food might increase the likelihood to notice food stimuli and be more responsive to these. Therefore, we argue that hunger as an evolutionary highly relevant physiological state might make certain stimuli goal-relevant and enhance motivation to pursue this goal; the value of such a stimulus might be as well enhanced in such a state and alter the threshold of conscious awareness. We, thus, hypothesized that the value of food stimuli is increased when the observer is hungry so that food stimuli have a higher probability to be noticed by hungry participants than by satiated participants.

The combination of food stimuli and hunger (i.e., craving) might be a perfect fit to investigate a potential general effect of value on inattentional blindness. In the present study, we extended the work of Li et al. (2015) in several ways: first, we used different food stimuli instead of only one specific type. Second, we also generalized the type of craving by choosing hunger as an independent variable instead of a food-specific craving. Third, we used a more controlled experimental design, as we randomly assigned our participants to two groups and actively manipulated the hunger level of our participants. Finally, we took all sexes into account. An advantage of the present design is that the unexpected object can be physically identical between the experimental groups. Also, we can investigate general cognitive effects of hunger using food-related but also non-food-related critical stimuli.

Since a specific type of food craving, namely the trait of ice cream craving, seems to modulate the probability of inattentional blindness (Li et al., 2015), we additionally investigated the effect of such craving traits. As we used different kinds of food as unexpected stimuli, we naturally assessed *general* food craving as a trait variable. This assessment will enable us to investigate whether food craving as a trait moderates the effect of hunger on inattentional blindness.

This study is of theoretical importance as it will expand our understanding of cognitive consequences of food deprivation and (more generally speaking) attentional orienting towards meaningful stimuli. This gained knowledge might pave the ground for a transfer into practice as advertisement for healthy foods or public awareness for involuntary attentional orienting towards food when hungry (e.g., in traffic).

## Methods

The collected and analysed data can be found as supplemental material (https://osf.io/zj5yg/?view_only=6548fde69a3a414181743098395f124b). The experiment was reviewed and approved by the ethics committee of the German Sport University Cologne.

### Participants

240 participants were tested at the German Sports University in Cologne. All participants gave written informed consent and received 5€ for their participation.

We excluded participants from analysis who indicated in a questionnaire that they expected the critical object or knew that inattentional blindness was the subject of this study (12 participants were excluded), participants who did not have normal or corrected-to-normal vision (4), participants who did not notice the unexpected object in the full-attention trial, when they were not distracted by the primary task (21), and participants whose data has been lost due to technical problems (1). All exclusion criteria were defined prior to data collection. Data of the remaining 202 participants were analysed (*M*_age_ = 22 years, SD_age_ = 3 years, 47% female, *M*_BMI_ = 22.38, SD_BMI_ = 2.25).

### Materials and procedure

The assignment of the participants to the two conditions (hungry, satiated) was counterbalanced prior to the experiment. Participants in the hungry-group (H-condition) were instructed to refrain from eating but continue drinking as usual (water or tea; no liquids and satiating drinks as milk, juice, or soda), for 16 h prior to the experiment. This length of time was chosen as Morris and Dolan (2001) have shown that food deprivation lasting 16 h leads to increased hunger ratings. Participants in the satiated group (S-condition) were instructed to eat as usual prior to the experiment. Participants were tested alone or in pairs. When tested in pairs, both working spaces were divided by room-dividers and participants were instructed to work quietly. The experiment lasted for approximately 15 min. Participants were seated at a distance of approximately 50 cm from a 24-inch screen (resolution: 1920 × 1080 pixels). First, they filled in paper–pencil versions of the German Food Cravings Questionnaire-Trait (FCQ-T-r), the German Food Cravings Questionnaire-State (FCQ-S), as well as the first part of the general questionnaire, including demographics and the perceived hunger on a visual analogue scales (VAS). Following this, they performed a computerized task, namely the static inattentional blindness task (adapted from Mack & Rock, 1998). This specific task was chosen as it is widely used in the inattentional blindness literature due to its standardized and controlled structure, its flexibility to create adaptations, as well as the ease of its application (see Redlich et al., 2020 for a review). Finally, the second part of the general questionnaire assessing knowledge about inattentional blindness was completed. Upon completion of all tasks and questionnaires, participants were debriefed. The debriefing encouraged participants to avoid sharing information about the experimental procedure with still-to-be-tested participants, as it is crucial that participants are unaware about their participation in an inattentional blindness study.

The computerized inattentional blindness task was programmed and run on Presentation 18.1 (Neurobehavioral System, Berkeley, CA). All instructions were delivered via a computer screen. Participants were encouraged to ask questions whenever they had not fully understood any of the instructions.

#### German Food Cravings Questionnaire-State (FCQ-S; Meule, Lutz, Vögele, & Kübler, 2012)

The FCQ-S is part of the general FCQ (Cepeda-Benito, Gleaves, Williams, & Erath, 2000) and assesses the current craving for a variety of foods from different categories (i.e. “I feel an intense desire to eat one or more specific foods”). The FCQ-S consists of 15 items for which individuals have to indicate on a 5-point Likert scale (1 = strongly disagree, 5 = strongly agree) to what extent they agree with each statement in the moment of completing the questionnaire (*right now, at this very moment*). The internal consistency for the FCQ-S total score in our sample was very high (Cronbach’s *α* = 0.95) and ranged between *α* = 0.88 (desire/ lack of control), *α* = 0.89 (reinforcement) and *α* = 0.90 (hunger) for the subscales. Furthermore, a validation study confirmed good psychometric properties for the German Food Craving Questionnaire (Meule et al., 2012).

#### German Food Cravings Questionnaire-Trait-reduced (FCQ-T-r; Meule, Hermann, & Kübler, 2014)

The FCQ-T-r is a short version with 15 items of the FCQ-T and assesses the frequency of food-craving experiences on a 6-point Likert scale (from 1 = never to 6 = always) (Cepeda-Benito et al., 2000). The items belong to five dimensions of the original FCQ- T: Control (items 2, 3, 25, 26, 29), Thoughts (items 6, 8, 27, 32, 33), Intent (items 5, 18), Emotions (items 20, 34), and Stimuli (item 36). The internal consistency of the FCQ-T-r total score in our sample was high (Cronbach’s *α* = 0.89). With regard to construct validity, the FCQ-T-r scores were weakly positively correlated with the Body Mass Index (BMI) and moderately negatively correlated with self-perceived dieting success (Meule et al., 2014). In line with results of validation studies of the long version (FCQ-T, Meule et al., 2012), the FCQ-T-r was positively correlated with attentional impulsivity, restrained eating, and eating disorder psychopathology (Cepeda-Benito et al., 2000; Meule et al., 2012).

#### General questionnaire incl. visual analogue scales

As recommended by Blundell et al. (2010), we used 115 mm line VAS, in which participants rated their appetite sensations (hunger, fullness, satiation, desire for a meal, and prospective food consumption). The five scales were anchored at the low end with the most negative feelings (e.g., very hungry) and opposing terms at the high end (e.g., not hungry at all). The overall score for *hunger perception* was based on the combination of the scores from the five VAS scales. Different studies have acknowledged VAS scores as reliable for appetite research (e.g., Flint, Raben, Blundell, & Astrup, 2000; Morris & Dolan, 2001). Furthermore, the general questionnaire assessed the personal preferences for the used food stimuli (“how much do you like”: burger, bread, chocolate) on a 6-point Likert scale (from 1 = very much to 6 = not at all). The general questionnaire also included questions about the last time participants’ had eaten, demographic information, knowledge about inattentional blindness, and the general motivation to participate in this study.

#### Static inattentional blindness task (adapted from Mack & Rock, 1998) including food-related pictures.

**Fig. 1:**
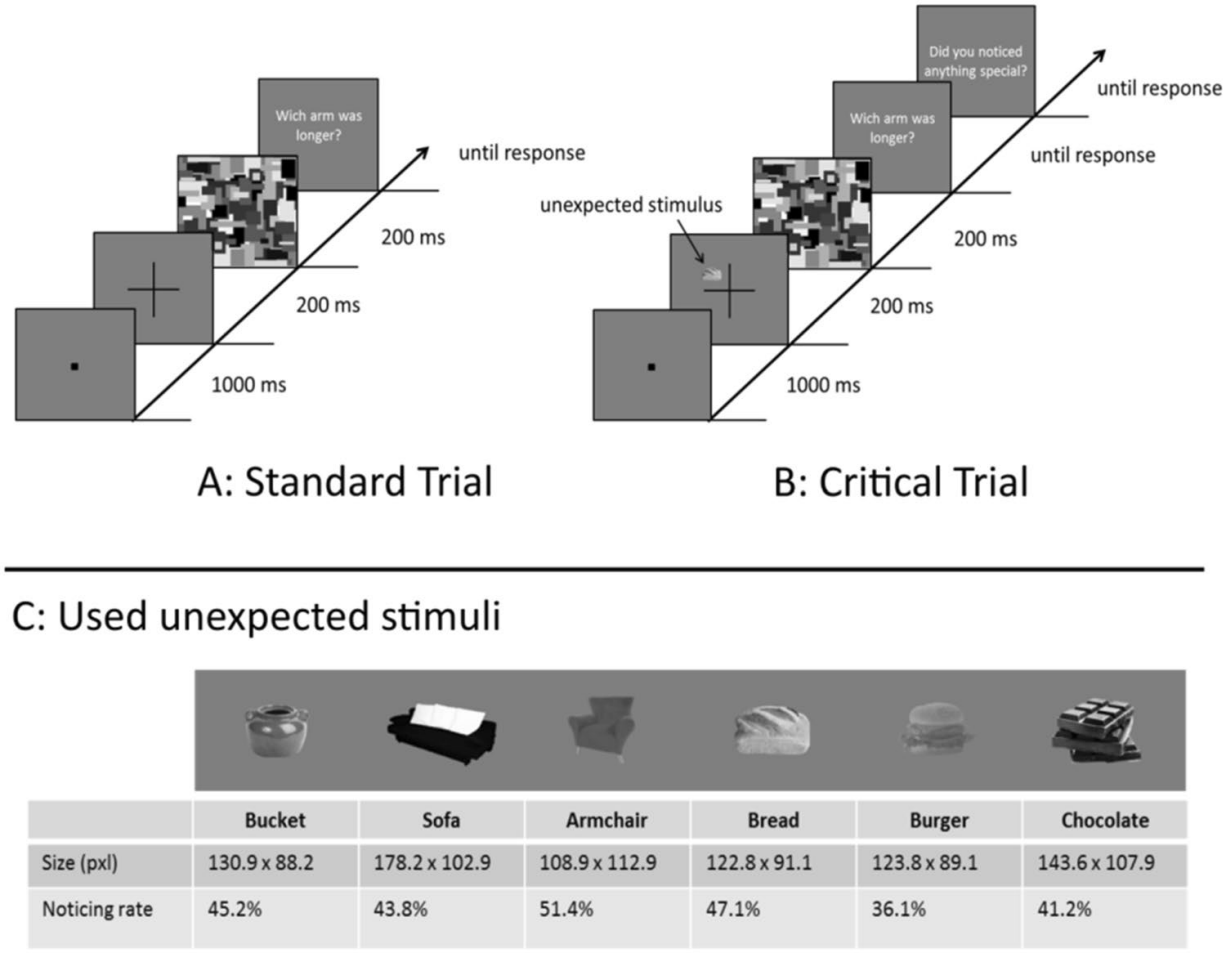
Schematic illustration of trials in the inattentional blindness phase (details not drawn to scale). **a** Standard trial during this phase, in which participants looked for the longer arm of the cross. **b** Critical trial, in which an unexpected object (food or furniture picture) appeared next to the to-be-attended cross. **c** Graphic representation, size and noticing rate of the used stimuli in the inattentional blindness task

The static inattentional blindness task consisted of 15 trials, of which the 11th trial was the critical, the 14th trial the divided-attention, and the 15th trial the full-attention trial. All remaining trials were labelled as standard trials. In each trial, participants were instructed to judge the length of the two arms of a briefly presented cross, placed at the centre of the screen and indicate by button press which arm was longer (Fig. 1a). On half of the trials, the horizontal arm of the cross was the longer one (189 pixels, corresponding to 6° visual angle), while the vertical arm was longer on the other half of the trials. These two types of trials (horizontal longer vs. vertical longer) were presented in random order. The shorter arm had a length of 150 pixels during the first five standard trials, which made it easy to discriminate it from the longer arm. On the remaining five standard trials, the shorter arm was 177 pixels long, which made it more difficult to discern the difference in length. Note that we chose to use 177 pixels because this was the length that resulted in 79% accuracy in a staircase procedure in the same inattentional blindness task in a previous study and, thus, constitutes medium difficulty (Kreitz et al., 2015).

Each trial started with a fixation point (6 × 6 pixels; 1000 ms), followed by the respective cross (200 ms), a pattern mask (200 ms; to prevent afterimages), and a response slide to remind the participants of the response-key mapping (no time limit). The 11th trial was the critical trial including the unexpected stimulus that was presented without forewarning alongside the cross for the entire 200 ms (Fig. 1b). Depending on the experimental condition, the unexpected stimulus was a black-and-white version of either a food or furniture picture and was derived from the Food-picture database (Blechert, Meule, Busch, & Ohla, 2014): pictures 0167 (chocolate), 0440 (bread), 0065 (burger), 1219 (armchair), 1059 (sofa), and 1217 (bucket). The specific choice of stimuli was based on different previous studies: (a) Koivisto, Hyönä, and Revonsuo (2004) found that stimuli are easier to be consciously detected in an inattentional blindness paradigm when they were coloured. Since all used stimuli are differently coloured, we used black-and-white versions of food and furniture stimuli to avoid any colour effects, but still activate a mental representation of the respective foods and furniture. Such mental representations should be strong enough for our manipulation, as even food words create mental representation strong enough to capture attention (Mogg et al., 1998). (b) Burger, bread, and chocolate were used as food stimuli since they were all rated high in *palatability* and *desire to eat* in a previous study (Blechert et al., 2014)^1^ and might also be seen as energy-dense foods which capture more attention than low-energy foods (Cunningham & Egeth, 2018). (c) To include a range of foods and taste, we used three different food pictures (Blechert et al., 2014). (d) To avoid floor or ceiling effects (i.e., insufficient variability in detecting the unexpected object as almost no one or everyone would see it independent of condition), which might conceal potential effects of our experimental manipulation, we adapted the contrast of the critical stimuli in pilot testing phases so that the noticing rate for each picture settled at approximately 50%. Based on their binary fashion, inattentional blindness paradigms can be seen as less sensitive for potential effects. Consequently, the importance of a well-balanced noticing rate and their necessary adjustment beforehand has been highlighted in previous inattentional blindness studies (Kreitz, Furley, Memmert, & Simons, 2016; Kreitz, Furley, Simons, & Memmert, 2016). Nevertheless, the chosen stimuli were all clearly recognizable as such. The size of all stimuli was approximately 130 pixels × 100 pixels (Fig. 1c) and their positions were picked randomly from four possible locations, corresponding to the four quadrants of the cross with a distance of 141 pixels from the midpoint of the cross (4.47 degrees of visual angle from the centre of the screen).

After having completed the line judgment, participants were asked if they had noticed anything on that trial that had not been presented before. Regardless of their answer, they were then asked about two characteristics of the unexpected stimulus: the location (upper right, lower right, upper left, lower left) and the shape of the unexpected stimulus (six choices; bread, chocolate, burger, bucket, sofa, and armchair). Participants were asked to guess in case they had not noticed anything additional. Afterward, participants performed two standard trials (12th and 13th) of the cross task without any critical object. The 14th trial was the divided-attention trial including the same unexpected object as during the critical trial. The questions concerning the characteristics of the unexpected stimulus were the same as those presented after the critical trial. The 15th trial was a full-attention trial in which the same critical stimulus was presented once more. However, on this trial, participants were specifically instructed to not judge the length of the two arms anymore, but rather focus on the whole screen. The questions concerning the characteristics of the unexpected stimulus were the same as those presented after the critical and divided-attention trial. This control trial allowed us to test whether participants could actually detect the food- or furniture-stimulus if they were not unexpected and attention was not directed elsewhere. Each participant performed the static inattentional blindness task only once. The randomly selected critical stimulus stayed the same during the critical trial, the divided-attention trial, and the full-attention trial for each participant. Thus, each critical stimulus (bread, chocolate, burger, bucket, sofa, and armchair) was presented to 40 participants.

### Data analysis

A statistical power analysis was performed to estimate the necessary sample size (G*Power 3.1.9.2, Germany). As a previous study reported medium effect sizes (Li et al., 2015), we aimed to detect at least medium effects (*w* = 0.30). With a power = 0.80 and an alpha = 0.05, the projected sample size needed for effects of *w* = 0.30 was approximately *n* = 108. Thus, we tested 240 participants (120 for both the H-condition, and the S-condition), leaving 202 for analysis after having applied the above-mentioned exclusion criteria.

In the static inattentional blindness task, participants were coded as inattentionally blind if they reported that they had not noticed the unexpected stimulus in the critical trial or if they claim to have seen it, but could not define the location or shape of the unexpected stimulus (Kreitz, Schnuerch, Furley, Gibbons, & Memmert, 2015).

Separate chi-square tests were used to investigate, whether participants’ hunger state (hungry vs. satiated) influences the noticing rates of food as well as furniture unexpected stimuli. In addition to chi-square tests, odds ratios (OR) with 95% confidence intervals are reported as standardized measures of effect size.

To ensure a successful hunger manipulation, we compared both groups (hungry vs. satiated) in regard to the *hunger* variable and the *lack of control* variable from the FCQ_S as well as in regard to the *subjective perception of hunger* variable and *last time they had a meal* variable from the general questionnaire. Mann–Whitney *U* tests were used to compare participants of both groups (hungry vs. satiated), as our data are based on ordinal Likert scales and a priori Shapiro–Wilk tests declined normality for the *hunger* variable from the FCQ_S (*W*(201) = 0.911, *p* = 0.001), the *lack of control* variable from the FCQ_S (*W*(201) = 0.963, *p* = 0.001), the *subjective perception of hunger* variable (*W*(201) = 0.914, *p* = 0.001) and the *last time they had a meal* variable (*W*(201) = 0.766, *p* = 0.001). The Benjamini–Hochberg procedure was used as a modified version of the Bonferroni correction to protect against an accumulating type 1 error (Benjamini & Hochberg, 1995).

Whereas the above-mentioned analyses were specified in advance, additional analyses were based on the results of our pre-specified analyses and were, thus, explorative in nature. Consequently, the following analyses were not adjusted by a Benjamini–Hochberg procedure and should be replicated in additional future samples. Specifically, our explorative analyses included the use of a Spearman correlation to test whether participants’ *subjective* hunger state was related to the noticing rate of an unexpected food stimulus under conditions of inattention. Furthermore, to test sex and individual food-craving trait as moderator variables for the effect of hunger on noticing rates, we used two separate binary logistic regression analyses with the interaction term of sex and hunger condition and the interaction term of individual food-craving trait and hunger condition as predictors, respectively, and noticing of the unexpected object in the critical trial as the dependent variable.

Additionally, a chi-square test explored the effect of hunger on an adjusted definition of the noticing rate of unexpected objects.

Finally, we were aware of the weaknesses of null-hypothesis testing, namely that such analyses cannot provide evidence for the absence of an investigated effect. Therefore, we additionally investigated our null findings with a Bayesian approach using JASP (The JASP Team, 2020); we reported Bayes factors to quantify the relative support for a null model over alternative models (Harms & Lakens, 2018).

## Results and discussion

**Fig. 2:**
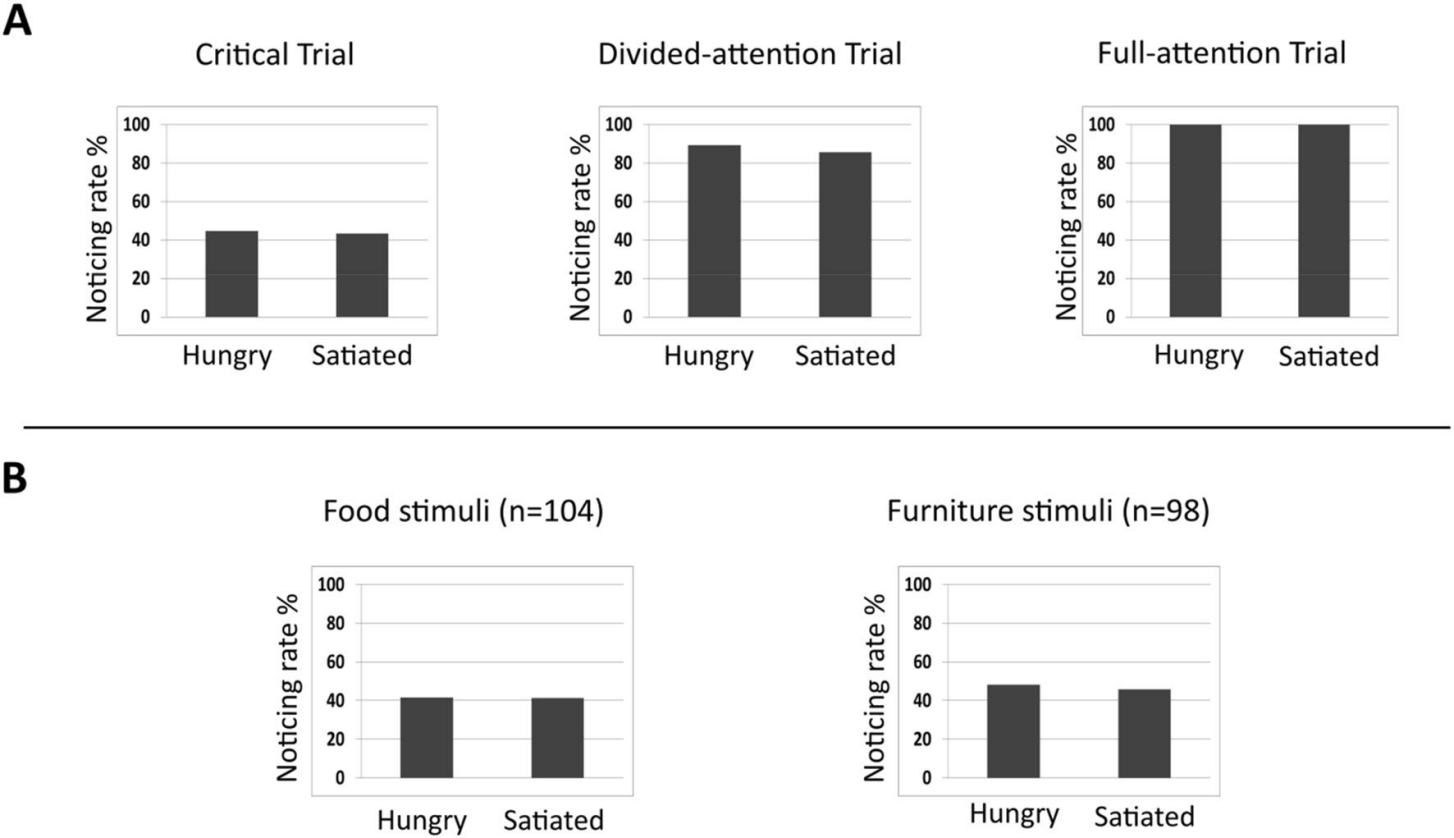
**a** Noticing rates of all unexpected stimuli (*n* = 202) in the critical trial, divided-attention trial, and full-attention trial separated by the experimental manipulation. **b** Noticing rates for unexpected food and furniture stimuli in the critical trial

The overall noticing rate in the critical trial was 44%. Thus, there was neither a ceiling nor a floor effect (Fig. 2a).

### Results and discussion of the inferential statistical analysis

We experimentally manipulated the food-craving state of participants to investigate whether hunger generates a higher value for food stimuli so that such stimuli are more likely to be noticed under conditions of inattention. The results showed that noticing rates for food stimuli did not differ between hungry (H-condition) and satiated (S-condition) participants [*χ*^*2*^(1) = 0.01, *p* = 0.981, RR(hungry/satiated) = 1.01 (95% CI: 0.46, 2.21)] (Fig. 2b).^2^ An additional Bayesian *χ*^2^ test revealed that it was approximately four times more likely that there was no effect than that hunger had an effect on inattentional blindness (*BF*_*01*_ = 4.22), which can be interpreted as substantial evidence (Kass & Raftery, 1995). Also, we were not able to support a more general effect of hunger using furniture stimuli as non-food-related critical stimuli [*χ*^*2*^(1) = 0.06, *p* = 0.972, RR(hungry/satiated) = 1.10 (95% CI: 0.49, 2.44), *BF*_*01*_ = 3.93]. Thus, hunger neither led to a lower threshold of awareness in general nor did it reduce the threshold of awareness for food-specific stimuli. This seems surprising in the light of previous findings suggesting an attentional advantage for valuable stimuli in inattentional blindness paradigms (as self-related stimuli; Mack & Rock, 1998, or evolutionary predetermined value; New & German, 2015). One might have thought that hunger would increase the value of food-related stimuli, as hunger is one of the most vital physiological functions.

In light of our null findings, the question arises whether participants followed our eating instructions and whether our manipulation was strong enough to actually implement the perception of hunger or saturation. To ensure a successful manipulation, we used Mann–Whitney *U* tests to compare participants of both groups in regard to subjective hunger perception, the last time they had a meal, and the *hunger* variable, as well as the *lack of control* variable of the FCQ_S. The results showed statistically significant differences between participants in the H-condition and participants in the S-condition on all measures, suggesting a successful manipulation (Table 1).

**Table 1:** Descriptive statistics and results of the Mann Whitney *U*-tests between the hunger and the satiated condition and descriptives for the respective variables

	Hunger (*n* = 105)		Satiated (*n* = 97)				
	Mean	SD	Mean	SD	*U*	*z*	*p*
Age	21.19	3.68	21.79	2.86			
BMI	22.28	2.02	22.49	2.48			
FCQ_T	40.13	10.26	36.82	9.46			
Subjective perspective of hunger	22.86	12.23	81.03	19.89	100.00	− 12.30	0.012^a^
fasted time	16.68	1.68	1.58	1.46	1.00	− 12.30	0.008^a^
FCQ_S hunger	11.98	1.86	5.30	2.10	192.00	− 11.86	0.008^a^
FCQ_S lack of control	19.19	3.88	10.86	3.26	594.00	− 10.82	0.008^a^

*p *significance (two-tailed), *BMI *body mass index, *FCQ_T *German Food Cravings Questionnaire-Trait, *FCQ_S *German Food Cravings Questionnaire-State

^a^*p *values are corrected with the Benjamini–Hochberg procedure as a modified version of the Bonferroni correction

### Results and discussion of the explorative statistical analysis

#### Different operationalizations of hunger

The results of our manipulation check showed that we can be quite confident that participants from the H-condition felt indeed hungrier at the time of testing than participants from the S-condition. Nevertheless, the perception of hunger is a very subjective concept with strong individual differences. Due to such individual differences and a lack of control in our experimental manipulation, as we completely depended on the cooperation of our participants, we cannot guarantee an optimal experimental manipulation.^3^ Therefore, a Spearman correlation between the subjective perception of hunger and the noticing rates of food stimuli in the critical trial was used to test (potentially in a more sensitive way than the binary experimental manipulation) the relationship between hunger perception and inattentional blindness. However, this alternative operationalization of hunger did not show a significant relationship with susceptibility to inattentional blindness, either (*ρ* = − 0.04, *p* = 0.694, *BF*_*01*_ = 6.48). Thus, hunger did not modulate the noticing rate of unexpected food stimuli independent of its operationalization as time of food deprivation or subjective hunger feeling.

#### Exploring different possible explanations for the null findings

Assuming that value was established via induction of hunger (see above), we did not find an effect of value of the unexpected object on the probability of its detection. This is consistent with Redlich et al., (2019) who found no significant effect of short-term learned monetary value on inattentional blindness. Nevertheless, our findings seem surprising as (A) previous research has shown a clear effect of hunger on attentional bias towards food stimuli (Morris & Dolan, 2001; Piech et al., 2010), (B) other studies have repeatedly shown that previously rewarded stimuli are preferentially processed and, thus, suggested that rewards are important in salience determination (Anderson, Laurent, & Yantis, 2011; Anderson & Yantis, 2012), and (C) noticing in an inattentional blindness paradigm has repeatedly been shown to be sensitive to other forms of value (attentional set: Most & Astur, 2005; Most et al., 2001; Koivisto & Revonuso, 2007; self-related stimuli: Mack & Rock, 1998, or evolutionary predetermined value: New & German, 2015).

Different explanations for these null findings seem reasonable. (A) One explanation might be the existence of moderator variables. For example, previous research suggests that sex might determine specific food craving: some food stimuli rich in carbohydrates as ice cream have been found to be the most regularly craved foods among females (Christensen & Pettijohn, 2001). Based on this, Li et al. (2015) already examined female participants with food-specific stimuli and food-specific cravings to investigate the effect of value on inattentional blindness. Potentially, cravings and, thus, the value of the food stimuli used in the present study were also higher for female than male participants as our food stimuli were rich in carbohydrates (chocolate, bread, and burger). To test this notion, we conducted a binary logistic regression analysis with the interaction term of sex and hunger condition as predictor and noticing of the unexpected food stimulus in the critical trial as dependent variable. Although we did not find a significant interaction effect for hunger condition and sex (*B* = − 0.63, SE = 0.32, Wald = 3.96, *p* = 0.071) on noticing, an additional chi-square test revealed that male participants were generally (i.e., independent of hunger condition) significantly more likely to notice unexpected food stimuli when their attention was engaged elsewhere (55%) than females (25%) [*χ*^*2*^(1) = 8.61, *p* = 0.003, RR(male/female) = 2.21 (95% CI: 1.29, 3.81), *BF*_*10*_ = 32.51]. In contrast, no significant effects were found for the noticing rates of non-food stimuli between males (53%) and females (40%) [*χ*^*2*^(1) = 1.08, *p* = 0.299, RR(male/female) = 0.22 (95% CI: 0.85, 2.02), *BF*_*10*_ = 0.53]. Despite these sex differences in regard to the detection of unexpected food stimuli, separate chi-square tests did not reveal significant effects of hunger for females [*χ*^*2*^(1) = 0, *p* = 1, RR(hungry/satiated) = 1 (95% CI: 0.38, 2.66), *BF*_*10*_ = 0.30], nor for males [*χ*^*2*^(1) = 7.17, *p* = 1, RR(hungry/satiated) = 0.99 (95% CI: 0.62, 1.59), *BF*_*10*_ = 0.32]. These exploratory results might function as an interesting starting point for future investigations.

(B) A further reason for our null findings might be the short time period we used to induce hunger and, thus, the value of food stimuli. Sixteen hours of food-deprivation can be easily achieved by missing out one meal as breakfast. Thus, differences in hunger state might be reflected on the VAS scales but might still not be strong enough to have practical implications for attentional orientation. However, this approach is commonly used in the field of hunger research (Evers et al., 2011; Mogg et al., 1998; Morris & Dolan, 2001) and previous research has shown attentional bias effects of hunger by even shorter experimental manipulations (6 h of fasting to implement hunger, Tapper, Pothos, & Lawrence, 2010). In contrast, studies focusing on more general aspects of cognition found equivocal results (see Benau, Orloff, Janke, Serpell, & Timko, 2014), which might demonstrate the complexity of short-term fasting on cognition. Fittingly, the general assumption that the concept of reward itself depends on a multitude of mechanism and determinants is supported by the literature on reward direction (gain vs. loss; Kahneman & Tversky, 1979) and the subjective reward value and probability (Chapman, Gallivan, & Enns, 2015). The manipulation chosen in the present study might be potent enough to have an impact on a sensitive measure as reaction times (Piech et al., 2010; Tapper et al., 2010), but not on the binary measure of awareness. Similar, such measure differences have also been found for priming effects (Kreitz et al., 2015) and monetary value (Redlich et al., 2019).

In contrast, other value-stimulus associations are based on long-term processes; meaningful and overlearned words (“Stop”, Mack & Rock, 1998) or threatening objects (spiders, New & German, 2015) have been found to successfully influence inattentional blindness. Potentially, including the personal food-craving trait into the analysis yields effects as it is also based on a long-term association process and might create a higher value for food stimuli. Consequently, we explored the individual food-craving trait as an additional variable that might modulate the relationship between hunger condition and noticing of the unexpected food stimulus in the critical trial. However, there was no significant interaction effect for hunger condition and individual food-craving trait on noticing [*B* = 0.01, SE = 0.01, Wald = 0.33, *p* = 0.567]. It seems that even general food craving as a trait is not strong enough to increase the food stimulus´ value and its likelihood to be noticed in an inattentional blindness paradigm.

(C) Another explanation for the null finding might be that in our study only 66% of the participants consciously perceived the shape of the unexpected stimulus when they said they noticed something in addition to the cross, whereas in contrast, everyone was able to choose the correct location. Potentially, the stimulus strength was not high enough for all participants to process the inherent meaning of the stimuli. We took this potential caveat into account and redefined noticing as “having noticed something in addition” and “being able to identify the correct shape”. However, there was no significant effect of hunger condition on the noticing of food stimuli [*χ*^*2*^(1) = 2.09, *p* = 0.148, RR(hungry/satiated) = 0.52 (95% CI: 0.22, 1.27), *BF*_*10*_ = 0.6] in this case, either.

(D) There might not be a general effect of hunger on the detection of food stimuli. That is, hunger might have an influence on attentional selection but this effect might not always be strong enough to be found in every paradigm. Previous studies showed that hunger leads to an increase in selective attention (Mogg et al., 1998), improves the memory advantage for food stimuli (Morris & Dolan, 2001), and limits attentional shifting (Piech et al., 2009). The only study so far showing direct effects of craving on the detection of unexpected food stimuli was the quasi-experimental study by Li et al. (2015) that investigated effects of ice cream craving on noticing ice cream stimuli. Building on these results, we aimed to prepare the grounds for a general effect of value of the unexpected object on inattentional blindness through the comprehensive inclusion of different sexes, different food stimuli, and an overall food craving in our study. However, we did not find any effect of value based on our experimental hunger manipulation, indicating that the effects of Li and colleagues might not easily be generalized. Admittedly though, these results should be treated with caution; additional Bayesian analyses only moderately supported our null findings in contrast to the alternative hypothesis.

#### Prospects: the general impact of value on inattentional blindness

With regard to the general inattentional blindness literature, our findings lead to the assumption that effects of value cannot simply be generalized. This is in line with other types of value as faces, whose effects have been investigated in the phenomenon of inattentional blindness; several studies found that faces were more likely to be noticed compared to other stimuli (Devue, Laloyaux, Feyers, Theeuwes, & Brédart, 2009; Lee & Telch, 2008; Mack & Rock, 1998) and argued that this effect is driven by the stimuli’s importance (Mack, Pappas, Silverman, & Gay, 2002). In contrast, Mack and Clarke (2012) showed that the presence of faces does not lead to higher noticing rates of an unexpected scene. Based on general assumptions about reward direction (gain vs. loss; Kahneman & Tversky, 1979), the subjective reward value (Chapman et al., 2015), and our failed attempt to extend a stimulus-specific effect of semantic value on inattentional blindness towards a more general effect of semantic value, we theorize that the concept of semantic value can probably be divided into subtypes, based on the values’ characteristics and underlying mechanisms. Specifically, we propose that the semantic value of a specific stimulus or stimulus group is based on certain characteristics.

The first characteristic is the length of time during which an object is associated with semantic value (long term vs. short term), so that semantic value created through a long-term learning process might be stronger compared to semantic value created through a short-term learning process. Thus, the semantic value of spiders and snakes (New & German, 2015) can be seen a strong, since it is learned in an evolutionary long-term process, whereas the semantic value of stimuli associated with monetary reward might be seen as weak since this was learned in a period of only 20 min (Redlich et al., 2019).

The second characteristic might be the quality of the association process, that is, whether it constitutes a high-relevance situation. Traumatic experiences might create a strong semantic value for stimuli associated with such a traumatic experience, whereas everyday experiences might not create a strong semantic value for stimuli associated with usual daily experiences.

The third characteristic could be the valence direction of the associated semantic value (positive vs. negative). For example, happy faces seem to be associated with stronger semantic value compared to frowning or sad faces (Lee & Telch, 2008; Mack & Rock, 1998).

The fourth characteristic that might influence the semantic value of a specific stimulus or stimulus group is the attentional set formed by context factors. The attentional set can be described as the “tuning” of one’s attention to prioritize certain features over others (Most, 2013) and, thus, strengthens the value of the prioritized features. Such “tuning” can be caused by environmental aspects; in a task we tune our attention to relevant stimuli that help us successfully perform this task, for example, triangular shapes or red stimuli in a computer task. In a traffic situation, our attention is more “tuned” to detect a human than to detect a kangaroo, as we might have experienced more men in business suits than kangaroos crossing a street in the city (Pammer & Blink, 2013).

We predict that a combination of these characteristics defines the semantic value of a specific object or event for a specific person in a specific context. Some characteristics, as the length of time during which a value association was learned, might apply to a large extent of the population, whereas others might only apply for a few. Therefore, we argue, that most value-driven attentional amplification might be sufficient to show in sensitive measures as reaction times (e.g., Mogg, et al., 1998; Redlich et al., 2019), but might not always suffice to help an object cross the threshold of awareness under conditions of inattention.

## Limitations

Certainly, our study is not free of limitations. First, the difference in ones’ subjective perception of hunger could also be caused by a potential hawthorn effect (Wickström & Bendix, 2000), so that participants overrated their hunger perception in response to their awareness of being observed and their knowledge about the studies’ content.

Furthermore, one might argue that the used black-and-white versions of each picture are less appealing than coloured ones and decrease the semantic value of the stimuli. Even though coloured pictures might have been an even better option, we believe that the used black-and-white food stimuli activate a mental representation of the respective foods, which, similar to food words (Mogg et al., 1998), capture attention and should (in combination with our experimental manipulation) establish a high value for food stimuli. The activated mental representations of each food stimulus can be supported by the data of the full-attention trial in which 95% of the participants correctly identified the food picture. However, one relevant challenge of the black-and-white pictures could be that they might not have been identified as energy-dense foods rated high in *palatability* and *desire to eat*, since the black-and-white picture of chocolate could be interpreted as dark and bitter chocolate and the black-and-white picture of bread could be interpreted as dark whole-grain bread. This might have weakened potential value effects since low-energy foods capture less attention than energy-dense foods (Cunningham & Egeth, 2018).

Another limitation might be the design of our hunger manipulation. Although previous studies have shown that 16 h of fasting leads to increased hunger ratings (Morris & Dolan, 2001), other manipulations included stricter diets. Furthermore, some participants might skip breakfast regularly while others are just not hungry in the morning. Thus, participants in the hunger group might easily meet the eating restrictions without changing their daily habits. Even though we included a manipulation check that confirmed the effectiveness of our design, the hunger manipulating might not have developed its full power.

Furthermore, one might consider to not only manipulate the hunger group but also the satiated group in future studies. Following previous studies, participants in the satiated group were instructed to eat as usual (Montagrin, Martins-Klein, Sander, & Mather, 2019; Piech et al., 2010). To ensure an even higher level of satiation, one might instruct the satiated group to eat specific meals prior to the experiment as it was done in other studies (Nederkoorn, Guerrieri, Havermans, Roefs, & Jansen, 2009; Mogg, Bradley, Hyare, & Lee, 1998).

Unavoidably, the attentional set of all our participants might have been biased towards food cues as they knew that they had to fast or eat as usual. An additional cognitive task prior to the inattentional blindness paradigm might help neutralize such an attentional set based in instructions in future studies.

Finally, it is important to mention that we calculated our sample size based on a previously found effect size in the related literature. This might be biased due to a publication bias, though; null findings are often not published (Murtaugh, 2002) and small sample studies tend to produce larger effect sizes than studies using large samples (Kühberger, Fritz, & Scherndl, 2014). Therefore, our power calculation might be based on an overrated effect so that we might have missed the smaller but real effect. Such considerations emphasize the importance to design experiments with appropriate sample sizes and to also publish null findings.

## Conclusion

Whereas other types of value based on evolutionarily predetermined relevance as a threat (New & German, 2015) or overlearned value as one´s name (Mack & Rock, 1998) affect the susceptibility to inattentional blindness, we did not find effects of value based on hunger and food stimuli. It seems that manipulations of value are not easily generalized. Possibly, different underlying mechanisms are involved for different types of value and some value manipulations might just not be strong enough to control whether or not an object crosses the threshold of awareness. Alternatively, effects might be too small to be detected in a design with binary outcome as the inattentional blindness paradigm. In any case, our findings indicate that the influence of on object’s value on conscious detection is not as clear as often suggested. Therefore, future research should focus on replicating previous effects of value and, additionally, make an effort to systematically define characteristics of different sources of value.

The benefits for future research are twofold: from a practical point of view, we strongly recommend focusing on a well-designed and powerful manipulation to create the value of interest. From a theoretical point of view, our findings help to expand knowledge on the factors that influence failures of awareness. Since the here used phenomenon of inattentional blindness is highly relevant for our everyday life (e.g., traffic or medical diagnostic) it seems important to study the conditions and factors that influence the likelihood of that phenomenon.

## Data Availability

The collected and analysed data can be found as supplemental material (https://osf.io/zj5yg/?view_only=6548fde69a3a414181743098395f124b).

## Footnotes

1 We included vegetarians in our study, as these ratings did not differ between vegetarians and omnivores.

2 The raw data can be accessed via https://osf.io/zj5yg/?view_only=6548fde69a3a414181743098395f124b

3 It was not possible to analyze extreme groups in our design as Li et al. (2015) did with 0.2 quantiles of subjective food craving. The sample size would be very small in such an analysis and reliable findings could not be expected.
